# Hospital Standardized Mortality Ratio: Consequences of Adjusting Hospital Mortality with Indirect Standardization

**DOI:** 10.1371/journal.pone.0059160

**Published:** 2013-04-09

**Authors:** Maurice E. Pouw, Linda M. Peelen, Hester F. Lingsma, Daniel Pieter, Ewout Steyerberg, Cor J. Kalkman, Karel G. M. Moons

**Affiliations:** 1 Department of Anesthesiology, University Medical Center Utrecht, Utrecht, The Netherlands; 2 Julius Center for Health Sciences and Primary Care, University Medical Center Utrecht, Utrecht, The Netherlands; 3 Department of Public Health, Erasmus Medical Center, Rotterdam, The Netherlands; 4 Department of Social and Behavioral Sciences, Tilburg University, Tilburg, The Netherlands; University of New South Wales, Australia

## Abstract

**Background:**

The hospital standardized mortality ratio (HSMR) is developed to evaluate and improve hospital quality. Different methods can be used to standardize the hospital mortality ratio. Our aim was to assess the validity and applicability of directly and indirectly standardized hospital mortality ratios.

**Methods:**

Retrospective scenario analysis using routinely collected hospital data to compare deaths predicted by the indirectly standardized case-mix adjustment method with observed deaths. Discharges from Dutch hospitals in the period 2003–2009 were used to estimate the underlying prediction models. We analysed variation in indirectly standardized hospital mortality ratios (HSMRs) when changing the case-mix distributions using different scenarios. Sixty-one Dutch hospitals were included in our scenario analysis.

**Results:**

A numerical example showed that when interaction between hospital and case-mix is present and case-mix differs between hospitals, indirectly standardized HSMRs vary between hospitals providing the same quality of care. In empirical data analysis, the differences between directly and indirectly standardized HSMRs for individual hospitals were limited.

**Conclusion:**

Direct standardization is not affected by the presence of interaction between hospital and case-mix and is therefore theoretically preferable over indirect standardization. Since direct standardization is practically impossible when multiple predictors are included in the case-mix adjustment model, indirect standardization is the only available method to compute the HSMR. Before interpreting such indirectly standardized HSMRs the case-mix distributions of individual hospitals and the presence of interactions between hospital and case-mix should be assessed.

## Introduction

In the last decades increasing attention is directed towards the quality of care of hospitals. Various performance indicators have been developed to express quality of care, among which the hospital standardized mortality ratio (HSMR). The HSMR is a risk adjusted hospital mortality rate that corrects crude hospital mortality rates by taking into account the case-mix of the hospital [1]. Developed and implemented in 1999, the HSMR is now used as a key hospital quality indicator in various countries including the United Kingdom, the United States, Canada, and the Netherlands [2], [3], [4], [5], [6], [7].

The HSMR is used by hospitals, health authorities, and media as a tool to assess the delivered quality of care, to analyse the trend of the quality of care of a hospital over time, and to compare and rank hospitals. Since its introduction, the HSMR has been debated for various reasons: the credibility of the link between quality of care and risk adjusted mortality [8], [9], [10], the variables that are used for case-mix adjustment [11], and issues regarding coding of these variables [12].

Another important, but often neglected issue, is the fact that the HSMR is computed via the so-called *indirect standardization* method. It has been long known that if mortality rates are adjusted via the indirect standardization method, these rates cannot always be compared [13], [14], [15], [16]. However, it seems almost inevitable that HSMRs of hospitals will be compared and ‘quality performance league tables’ will be constructed.

The present paper illustrates the potential pitfalls of HSMR when used to compare hospitals. We will first provide a description of the indirect and direct standardization method to demonstrate why caution must be taken when hospitals are compared and ranked based on indirectly standardized figures like the HSMR. Subsequently, we illustrate the consequences of indirect standardization in practice using HSMR figures from the Netherlands.

## Methods

### Ethics Statement

To study the impact of this phenomenon caused by indirect standardization on real clinical data, we have conducted a series of analyses on the Dutch HSMR figures, permitted by the Dutch Hospitals Association and the Dutch University Medical Centers Association. The data were obtained from the Dutch National Medical Registration database, which contains routinely collected hospital episode statistics of Dutch patients and is held by Dutch Hospital Data. All data were analyzed anonymously (http://www.dutchhospitaldata.nl/Bestanden/Documenten/Protocol_gegevensgebruik_DHD_databanken.pdf).

### Standardization methods

Differences in crude mortality rates between hospitals are not only caused by differences in hospital performance but also by differences in the case-mix of patients that are admitted. A hospital that admits on average older patients and performs a larger proportion of ‘high risk’ procedures is likely to have a higher in-hospital mortality rate than a hospital with on average younger patients and a smaller proportion of ‘high risk’ procedures. Standardization methods use information at patient level such as reason of admission, age, sex, deprivation category and comorbidity to adjust for these differences in case-mix.

A standardized mortality ratio is calculated as the observed number of deaths divided by the expected number of deaths. For the HSMR, this is the observed and expected mortality for a given hospital in a given year, expressed as a percentage. If the observed number of deaths is 120 and the expected number of deaths is 100, the HSMR for that hospital would be 120. A HSMR greater than 100 reflects more deaths than expected and a HSMR less than 100 reflects fewer deaths than expected.

There are two main methods of standardization: direct and indirect. The main difference between these two methods is *what* is being standardized, whether it being the case-mix (direct standardization) or the mortality rate (indirect standardization).

#### Direct standardization

The direct standardization method standardizes the case-mix of patients admitted in a hospital to a *reference* case-mix. A directly standardized mortality rate of a hospital is therefore based on the same case-mix as the directly standardized mortality rates of other hospitals, i.e. on the *reference population (reference case-mix)*. In this way the effect of differences in case-mix populations between hospitals is eliminated.

Directly standardized mortality rates are computed as follows. First, the probability of in hospital death is calculated for each subcategory of patients as number of deaths divided by the number of admissions in that subcategory. Thus for example the probability of in hospital death for men, treated for the diagnosis of pneumonia, in the age category 60–64 year, may be 2% in one hospital, whereas patients with the same combination of predictors may have a mortality probability of 3% in another hospital.

Secondly, the mortality probabilities of each hospital are applied to the same reference hospital population to obtain the expected number of deaths *in the reference hospital population*. If the reference hospital population has 100 patients in the subcategory of our example, the expected number of deaths according to the mortality rates of our example hospital would be 2 (100×2%). Summation of the expected number of in-hospital deaths of all subcategories gives the total expected number of in-hospital deaths in the reference hospital population. The ratio between the expected number of in-hospital deaths and the actual number of in-hospital deaths *in the reference population* gives the directly standardized mortality ratio for the hospital of interest. Note that a hospital must have patients in a subcategory to calculate the corresponding mortality rate for that subcategory, and that the number of patients in the subcategory must be large enough to obtain reliable mortality rates.

#### Indirect standardization

The indirect standardization method standardizes the mortality rate of the case-mix to a *reference mortality rate* (expected mortality rate). An indirectly standardized mortality rate of a hospital is based on the expected mortality rate for that hospital given its case-mix of patients.

The indirect standardization method calculates the expected number of deaths for a hospital in two steps. First, *expected* probabilities of in-hospital death are computed using a logistic regression model with in-hospital mortality (yes/no) as outcome and various patient characteristics as predictors. For this modelling one commonly uses the data of many (preferably all) hospitals in a particular country. These expected probabilities of in-hospital death are computed for each subcategory of patients and can be interpreted as the probability of in-hospital death for patients belonging to the corresponding subcategory in a *standard* hospital of that country. For example the prediction model might calculate that the expected in-hospital probability of death for men, treated for the diagnosis of pneumonia, in the age category 60–64 year is 3%.

Secondly, these expected probabilities are applied to the admission numbers of a specific hospital to compute the expected number of deaths *in that hospital*. If the hospital under study has admitted 200 patients in the subcategory of our example, the expected number of deaths in this subcategory would be 3% of 200 or 6 deaths. The summation of the expected number of deaths in all subcategories gives the total expected number of deaths for that hospital. The observed number of deaths in a hospital is calculated by simply counting the number of people who died *in the specific hospital* within the given period. The ratio between the observed number of deaths and the expected number of deaths gives the indirectly standardized mortality ratio.

The advantage of directly standardized mortality rates is that these rates are comparable with each other because the effect of differences in case-mix is eliminated, as they are all based on the same reference hospital population. However, a subcategory of patients of a hospital under study may be very small, resulting in an unreliable mortality rate (e.g. a mortality rate of 0% in a subcategory containing 5 patients). Moreover, if a hospital does not have any patients in a subcategory, the direct standardization method cannot be used at all. Therefore, in most cases, direct standardization is not applicable and the indirect standardization method is used. This is also the case for the HSMR as it is generally calculated. The drawback of indirectly standardized mortality rates is that these rates are not always comparable with each other as will be explained in the example below.

### Numerical example

We assume there are two hospitals, hospital A and hospital B, of equal size (both admitting 5000 patients per year) and both delivering precisely the same quality of care. If the HSMR is a fair and valid measure of quality of care, this measure should then also be equal for both hospitals. For reasons of simplicity, we distinguish only two kinds of patients (i.e. using one patient characteristic instead of the 9 normally used to calculate the expected mortality): urgently versus non-urgently admitted patients. Suppose that hospital A has admitted 20% of the 5000 patients urgently and hospital B 80%.

We now assume that urgently admitted patients have an *expected* mortality rate of 6% and non-urgently admitted patients of 2% (see also table 1). Furthermore, assume that in both hospitals the *observed* mortality rates for these groups are 3% and 4% respectively. Although a patient admitted to hospital A has the same chance to die as in hospital B (3% if admitted urgently and 4% if admitted non-urgently) the HSMR is 136 for hospital A and 61 for hospital B (table 1).

**Table 1:** Numerical Example of direct and indirect standardization.

	Hospital A		Hospital B	
	Urgent	Non-urgent	Urgent	Non-urgent
**Expected mortality rate**	6%	2%	6%	2%
**Observed mortality rate**	3%	4%	3%	4%
**Case-mix**	1000	4000	4000	1000
**Indirect standardization**	136		61	
	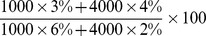		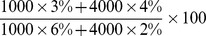	
**Direct standardization**	100		100	
	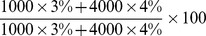		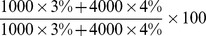	

Although both hospitals have the same observed mortality hospital A performs worse than hospital B when the mortality rate is adjusted via the indirect standardization method.

The difference in indirectly standardized HSMR is the result of the difference in case-mix and of interaction between case-mix and hospital and is explained as follows.

**Figure 1:**
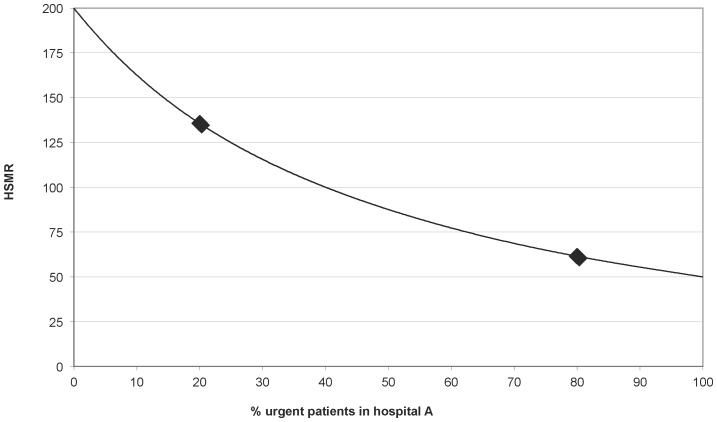
Change in HSMR when the ratio of urgently vs. non-urgently admitted patients changes. The observed mortality rates are 3% and 4% for respectively urgently and non-urgently admitted patients. The expected rates are 6% and 2%, implying the presence of statistical interaction between hospital and urgency, which is ignored in the adjustment model. Markers indicate the proportions of urgently admitted patients used in the theoretical example (20% and 80% respectively).

In both hospitals, urgently admitted patients have a lower probability to die than non-urgently admitted patients (observed rates 3% vs. 4%). In the total population the effect of urgency is the other way around (expected rates 6% vs. 2%). This means that there is statistical interaction between hospital and urgency. Although the two hospitals in the example perform similar, hospital B benefits from this situation as the majority of its population consists of urgently admitted patients (80% of the total), resulting in a lower HSMR than for Hospital A. Thus, when comparing and ranking the performance based on the single HSMR statistic only, hospital B is considered to be better than hospital A, despite the fact that the chance to die for a random patient is equal in both hospitals. Figure 1 displays the relation between the case-mix distribution (urgent – non-urgent ratio) and the HSMR, keeping the expected and observed mortality rates constant.

The direct standardization method is not affected by this present interaction. Suppose hospital A is the reference hospital. Then the case-mix of hospital B will be standardized to the case-mix of hospital A. Because the observed mortality rates for urgently and non-urgently admitted patients do not differ between the hospitals, the directly standardized mortality rates for hospital B and for hospital A are both 100 (see table 1).

Besides the use of the HSMR for comparisons across hospitals, it is also advocated to compare the HSMRs of a single hospital over time as an indicator of change in quality of care. However, the same phenomenon as described above can be found. When the case-mix distribution changes over time and interaction between hospital and case-mix is present, the HSMR can still change even if the quality of care (expressed as observed mortality rates) and the predicted risk for each patient remains constant. A worked-out example can be found in Appendix S1.

### Application to Dutch HSMR figures

To study the impact of this phenomenon caused by indirect standardization on real clinical data, we have conducted a series of analyses on the Dutch HSMR figures, permitted by the Dutch Hospitals Association and the Dutch University Medical Centers Association. For the present analyses, patient consent was not necessary as the data was stored and thus used completely anonimized. For the same reason, approval of a medical ethics committee was not needed.

The Dutch HSMR have been calculated in a similar manner to that used in several other countries and was performed by the authors (notably DP) in close collaboration with Dr Foster Intelligence (London, UK). For a detailed description of the Dutch HSMR models and used method, we refer to a previous publication [2]. In short: 50 diagnostic groups were selected which accounted for 80% of in-hospital mortality. For each diagnostic group a prediction model (logistic regression model) was fitted using various predictors, including age, gender, urgency of admission, month of admission, Charlson Comorbidity Index, diagnosis, and social deprivation, to generate an expected mortality risk for each admitted patient. In total, 4,031,829 admissions in the period 2003–2009 were included to fit these 50 prediction models. The HSMR is the sum of the observed mortalities in all 50 diagnostic groups divided by the sum of all expected mortalities. The coefficients of the predictors of the final models can be shown on request.

For the present analysis, we tested whether there was an interaction beween hospital and urgency of admission, as we hypothesized that the effect of the variable ‘urgency of admission’ on the outcome (death) might differ across hospitals. For example, high-level trauma centres are probably more adequate in treating acutely admitted patients. For each of the 50 diagnostic groups, we fitted a logistic regression model with the variables ‘hospital’, ‘urgency of admission’ and their interaction term ‘hospital*urgency of admission’ as predictors and tested whether the interaction term was significant (P<0.05). We repeated this analysis to test for interaction between hospital and comorbidity.

We analysed the HSMR of 61 Dutch hospitals from the period 2006–2009. For each hospital, we first calculated the HSMR according to the regular indirect standardization method. Then we analysed eight scenarios.

In the first part (scenario 1–4) we stratified patients according to their admission status into urgent and non-urgent admissions to mimic the numerical example as described above. For both groups, the size of the group, the observed mortality rate, and the expected mortality rate were calculated. Subsequently, we kept the observed and expected mortality rates for these two groups constant, and replaced the original distribution of urgent and non-urgent admissions for each hospital by the ‘average case-mix distribution of all 61 Dutch hospitals’ (scenario 1). The obtained HSMRs reveal what the HSMR of the hospital would have been if that hospital had an average Dutch hospital distribution of urgent and non-urgent admissions. To investigate more extreme variations, we extended these scenarios by replacing the original distribution of a single hospital by the case-mix distribution of a single other hospital (scenario 2).

In a third scenario we looked at the effect of differences in case-mix distributions of a single hospital on the HSMR over time. We used the observed and expected mortality rates of the urgent and non-urgent admissions of a hospital in the year 2009 as a basis. For each hospital, we recalculated the HSMR for 2009 using the hospital's average case-mix distribution of urgent versus non-urgent admissions over the years 2006–2009 (scenario 3). Finally, we recalculated the HSMR of each hospital with the distribution of urgent versus non-urgent admissions of the years 2006, 2007 and 2008 separately. Here, differences in HSMRs are then solely to be attributed to differences in distribution between urgent and non-urgent admissions over time (scenario 4).

We repeated these scenario studies using another case-mix variable ‘Charlson Comorbidity index’ (CCI) instead of ‘urgency of admission’ (scenario 5–8). The CCI is used as a score for comorbidity and is based on 17 comorbidities such as cancer, congestive heart failure, cerebral vascular disease, peripheral vascular disease, dementia, diabetes, and renal disease [17], [18]. Each comorbidity is assigned a weighted score. Depending on the patient's sort and number of comorbidities the CCI stratifies the patient into a class ranging from 0 (no comorbidity) to 6 (severe comorbidity).

## Results

**Figure 2:**
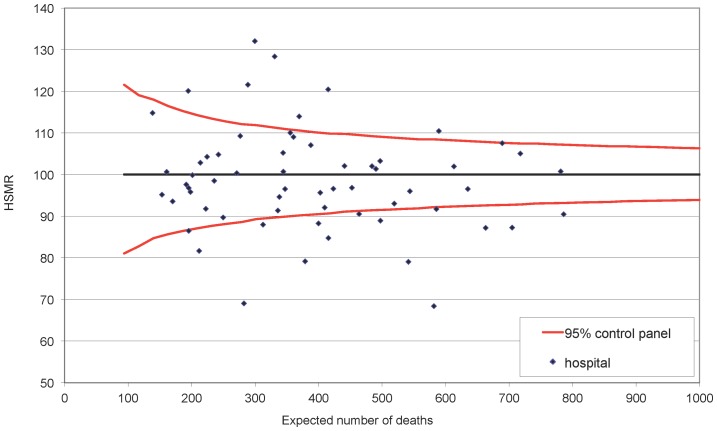
Funnel plot showing the HSMRs of Dutch hospitals in 2009.

Figure 2 shows a funnel plot of the HSMRs of the 61 hospitals. The funnel plot divides hospitals in three categories using 95% control limits. The 95% control limits demarcate the 95% confidence interval of the HSMR given the expected mortality. Hospitals above the 95% control limits have a HSMR significantly higher than 100, hospitals below the 95% control limits have a HSMR significantly lower than 100, and for hospitals between the 95% control limits a deviation from the reference value of 100 is considered to be a result of natural random variation. As can be seen in figure 2, in 2009 the HSMRs of Dutch hospitals differed considerably. Fifteen hospitals appeared to perform significantly better, and seven hospitals significantly worse than expected. According to the risk adjustment model used, the risk of dying in the hospital with the lowest HSMR is 1.93 times lower than the hospital with the highest HSMR (132/68).

**Figure 3:**
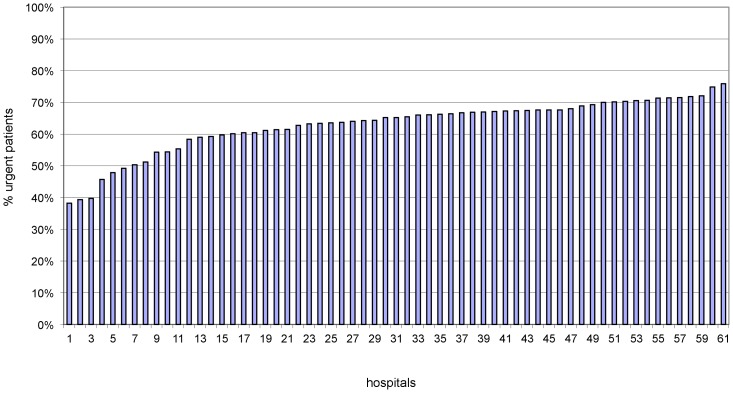
Proportion of urgently admitted patients per hospital (for the 50 CCS diagnoses used in calculating HSMR).

**Figure 4:**
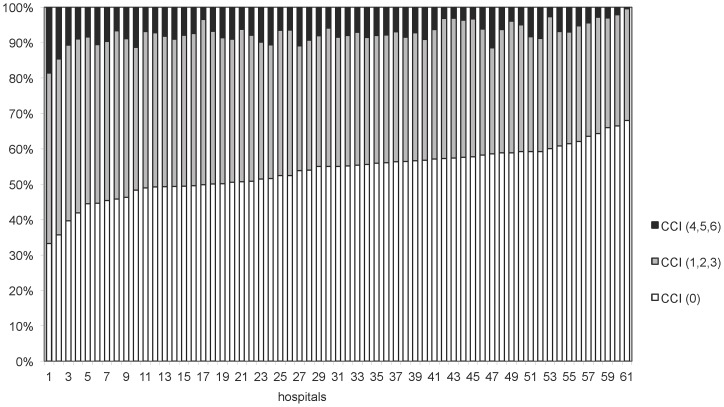
Distribution of the Charlson Comorbidity Index of patients per hospital (for the 50 CCS diagnoses used in calculating HSMR). Distribution of the Charlson Comorbidity index across hospitals. In this figure the Charlson Comorbidity Index groups 1,2 and 3 are aggregated as well as the groups 4,5, and 6.

In 2009 the 61 hospitals had 492,099 admissions of which 301,916 admitted urgently. Figure 3 illustrates the percentage of urgently admitted patients per hospital in 2009, which ranged from 38% to 76% (median 65% (IQR: 60%–68%). Respectively 53%, 20%, 15%, 5%, 1%, 5%, and 1% of the studied admissions were classified in the CCI group 0, 1, 2, 3, 4, 5, and 6. Figure 4 illustrates the distribution of the CCI of admissions per hospital in 2009.

We tested for interaction between hospitals and two case-mix variables: ‘urgency of admission’ and CCI. We found evidence of interaction between hospitals and ‘urgency of admission’ in 19 of the 50 prediction models (statistically significant interaction term, P<0.05). In 7 of the 50 prediction models we found evidence of interaction between hospitals and CCI.

For each hospital we recalculated the HSMR using the mean of the distributions of the case-mix variable ‘urgency of admission’ of the 61 hospitals (scenario 1). The relative change between these obtained simulated HSMR and the original HSMR ranged from −2.6% for one hospital (HSMR: 110, simulated HSMR: 107) to 5.5% for another hospital (HSMR: 79, simulated HSMR: 83). No hospital changed significantly from category (i.e. the same fifteen and seven hospitals respectively over- and underperformed according to the new computed HSMR). Appendix S2 shows the HSMR and the HSMR of scenario 1 for all hospitals. The top 5 and bottom 5 hospitals remained unchanged when the HSMR of scenario 1 was used to rank the hospitals (Table 2). The total absolute difference in HSMR was 44 points (an average of 0.72 points per hospital). Tables 3 and 4 show an overview of the results of the eight scenarios. In scenario 2, where we replaced the case-mix distribution of a hospital with the distribution of one single other hospital, we found that for 10 (16.4%) hospitals another hospital could be found whose case-mix distribution significantly changed the category in the funnel plot. For 7 of these 10 hospitals, the HMSR was close to a control limit (less than 2 HSMR points). In scenario 3 we replaced the case-mix distribution of a hospital in 2009 with the average case-mix distribution of that hospital (2006–2009). No hospital changed significantly from category with the new HSMR. The difference between the HSMR of scenario 3 and the original HSMR ranged from −4.0% for one hospital (the HSMR decreased from 69 to 66) to 0.8% for another hospital (HSMR of 79 increased to 80). The total absolute difference in HSMR was 19 points (an average of 0.32 points per hospital). In scenario 4 we replaced the case-mix distribution of a hospital in 2009 with the distribution of that hospital of a single previous year (2006, 2007 and 2008). For one hospital the distribution of year 2008 significantly changed the category in the funnel plot (from better than expected to average). However, the HSMR only changed one point (from 86 to 87). No hospital changed categories when the distribution of 2007 was used and one hospital changed categories when the distribution of 2006 was used (from worse than expected, HSMR of 110, to average, HSMR of 108).

**Table 2:** Top and bottom 5 hospitals based on their HSMRs.

Original			Scenario 1				Scenario 5			
Ranking	HSMR		HSMR-1		New ranking	Rank difference	HSMR-5		New ranking	Rank difference
1	68	(*)	69	(*)	2	−1	69	(*)	1	0
2	69	(*)	68	(*)	1	1	73	(*)	2	0
3	79	(*)	83	(*)	5	−2	77	(*)	4	−1
4	79	(*)	79	(*)	3	1	76	(*)	3	1
5	82	(*)	82	(*)	4	1	82	(*)	5	0
57	120	(**)	121	(**)	58	−1	114	(**)	53	4
58	120	(**)	120	(**)	57	1	117	(**)	54	4
59	122	(**)	125	(**)	59	0	123	(**)	56	3
60	128	(**)	128	(**)	60	0	129	(**)	59	1
61	132	(**)	132	(**)	61	0	155	(**)	61	0

The HSMR of scenario 1 is computed based on the mean of the case-mix distributions of the ‘urgency of admission’ variable of the 61 hospitals. The HSMR of scenario 5 is computed based on the mean of the case-mix distributions of the ‘Charlson Comorbidity index’ variable of the 61 hospitals. (*) Significantly lower than 100, (**) significantly higher than 100.

**Table 3:** Scenarios using an average hospital case-mix distribution.

		Number of hospitals changing ranks			
Scenario	Number of hospitals changing category	No change	1–5 ranks	5–10 ranks	>10 ranks
1	0	14	47	0	0
3	0	38	22	1	0
5	8 (3)	10	41	5	5
7	3 (1)	14	42	4	1

In scenario 1 and 5 the mean distribution of the case-mix variable under study of the 61 hospitals is used to recalculate the HSMR of the hospitals. In scenario 3 and 7 the HSMR of a hospital is recalculated using the mean distribution of the case-mix variable under study over time (2006–2009). In the second column the numbers of hospitals are shown for which the recalculated HSMR crosses a ‘control limit’. In brackets: the number of hospitals for which the HSMR lies close to a control limit (within 2 HSMR points). Columns 3 to 7 show an overview of rank changes of hospitals based on the recalculated HSMR.

**Table 4:** Scenarios using a unique hospital case-mix distribution.

		Significant HSMR change				
Scenario	Number of hospitals changing category	By 1 hospital	By 2–5 hospitals	By more than 5 hospitals	By 1 other year	By more than 1 year
2	10 (7)	2	3	5	N.A.	N.A.
4	2 (2)	N.A.	N.A.	N.A.	2	0
6	35 (13)	6	4	25	N.A.	N.A.
8	3 (1)	N.A.	N.A.	N.A.	0	3

In scenario 2 and 6 the HSMR is recalculated using the distribution of the case-mix variable under study of a single hospital. In these scenarios for each hospital 60 HSMRs are recalculated. In scenario 4 and 8 the HSMR is recalculated using the distribution of the case-mix variable under study of another year. In these scenarios for each hospital three HSMRs are recalculated. In the second column the numbers of hospitals are shown for which the recalculated HSMR crosses a ‘control limit’. In brackets: the number of hospitals for which the HSMR lies close to a control limit (within 2 HSMR points). Columns 3 to 5 show an overview of the number of hospitals whose case-mix distribution changes the HSMR of a hospital significantly. Columns 6 and 7 show an overview of the years where the differences in case-mix distribution change the HSMR of a hospital significantly.

When repeating the scenarios based on the Charlson Comorbidity Index (scenarios 5–8), differences between the original and simulated HSMRs increased. The relative change between the obtained simulated HSMR based on the mean co-morbidity distribution and the original HSMR ranged from −6.7% for one hospital (HSMR: 97, simulated HSMR: 90) to 29.5% for another hospital (HSMR: 95, simulated HSMR: 123). Eight hospitals, including the 2 hospitals mentioned above, changed significantly from category when the HSMR of scenario 5 was compared with its original HSMR. For seven of these eight hospitals the difference in HSMR ranged from −4 points to 1 point. The hospital with the largest HSMR difference (from 95 to 123) tumbled in the ranking list from position 23 to position 57. The HSMRs of the hospitals are shown in Appendix S2. The top 5 hospitals remained unchanged when the HSMR of scenario 5 was used to rank the hospitals, but the differences were larger in the bottom 5 hospitals.

Looking at changes over time, we found that the relative change between the HSMR with the average distribution of that hospital of the years 2006–2009 and the original HSMR ranged from −18.6% (HSMR dropped from 97 to 79) to 4.8% (HSMR increased from 97 to 101). Three hospitals significantly changed to another category because of a change in HSMR.

## Discussion

The HSMR is considered to be an important tool in the assessment of quality of care across hospitals as well as for a single hospital over time. In a growing number of countries the HSMR is used by hospital board members, the health inspectorate, media, and public to monitor and compare the quality of care. Given the fact that the HSMR is based on indirect standardization, it has been known – although in practice largely ignored – that such comparisons are only allowed if the underlying case-mix distributions are identical or if there is no interaction between hospitals and case-mix variables [13]–[16]. In this paper we showed the pitfalls of indirect standardization of the HSMR by means of a numerical example, and in practice by using Dutch clinical data.

With the numerical example we illustrated that when there is interaction between hospital and case-mix, the indirectly standardized HSMR is not only determined by the observed and expected mortality rates, but is also related to the distributions of the underlying case-mix variables. Thus, caution must be taken not only when interpreting and comparing HSMRs of different hospitals but also when comparing HSMRs of a given hospital over time. When there is no interaction between hospital and case-mix, direct and indirect standardization will lead to the same HSMR, also when there are differences in case-mix distribution.

From our empirical studies we learned that although changing the case-mix distribution of a hospital results in a different HSMR, differences between the HSMR calculated with the average Dutch case-mix distribution of the variable ‘urgency of admission’ (scenario 1) and the original HSMR, were small. In terms of ranking, we see little movement, and no hospitals moved across categories. However, replacing the hospitals' case-mix by that of a single other hospital (scenario 2) led to significant changes in HSMR and rank. Comparing the HSMR of a hospital with the HSMR computed with the average case-mix distribution of previous years of that hospital for the variable ‘urgency of admission’ (scenario 3), showed again only small differences in final hospital ranking. This is probably because the case-mix distribution of this variable did not change substantially over the last 4 years for the Dutch hospitals. From these three scenarios we learned that the larger the difference between the ‘original’ and the ‘new’ case-mix, the larger the change in HSMR will be due to the indirect standardization method itself.

The findings are also strongly dependent on which case-mix variable is investigated. For the variable ‘Charlson Comorbidity index’, the distribution differences between hospitals cause more discrepancy between the original HSMR and the simulated HSMR (scenario 5) than the simulation study in which the case-mix variable ‘Urgency of admission’ was used (scenario 1). This is probably due to more variability in the distribution of the Charlson Comorbidity index between hospitals. Nevertheless, the ranking of the top and bottom 10 hospitals still hardly changed. Furthermore, for some hospitals the distribution of the variable ‘Charlson Comorbidity index’ also varied considerably between years (scenario 8).

Some limitations of our study must be taken into account. We only looked at two of the nine case-mix variables used in the case-mix correction model. Moreover, in our scenario studies we varied only one variable at a time. Despite the high possibility that the distributions of the other case-mix variables also differ between hospitals and over the years, we explicitly chose to focus on these two variables because our goal was to provide insight in the fact that case-mix differences between Dutch hospitals can influence the HSMR and because these two variables are subject to debate in terms of coding issues [12]. Therefore, distribution differences between hospitals may distort the comparison of HSMRs more than revealed with this study. However, it might be expected that differences in the distribution of other predictors in the adjustment model would have similar effects. Another limitation is that we only looked at a period of 4 years (2006–2009). Although our study indicates that differences in case-mix distributions of hospitals over time do not influence the HSMR noticeably, it is very well possible that over a longer period of time case-mix distributions change, such that long term trend monitoring using the HSMR may be misleading.

Due to their indirect standardization, HSMRs may not automatically be comparable neither across hospitals nor for a single hospital over time, unless the underlying case-mix distributions are proportionally the same or when there is no interaction between hospital and case-mix. In our data we found evidence of interaction between hospitals and the case-mix variables ‘urgency of admission’ and ‘comorbidity’. However, our empirical example showed that when differences in case-mix were limited (scenario 1 and 3), the effect on the HSMRs and thus the ranking of the hospitals is limited. Only replacing a hospital's case-mix with a very different case-mix (scenario 6) led to significant changes in HSMRs. More importantly, direct standardization is practically impossible when multiple predictors are included in the adjustment model. The numbers of patients in each subcategory then become too small to obtain reliable mortality rates. Furthermore, it has been previously argued that the indirectly standardized HSMR provides the mortality rate from a societal perspective as it is based on the population the hospital actually serves, not the national reference population, while a HSMR based on direct standardization is more relevant to informing patient choices [19].

Although still subject to much discussion, HSMR will likely remain as one of the indicators for hospital quality. HSMRs should be interpreted, however, with the greatest caution, due to issues concerning the link between in hospital mortality and quality of care, coding differences between hospitals, insufficient case-mix adjustment, and poor data quality. In addition, in this study we have shown that the indirect standardization method used to compute the HMSR might also distort the interpretation of HSMRs. Therefore, we urge researchers to first investigate the distributions of the underlying case-mix variables and assess the presence of interactions between hospital and case-mix. A possible solution might be to analyse hospitals within clusters with comparable case-mix distributions such as small regional hospitals, large teaching hospitals and academic hospitals. Comparing HSMRs of hospitals belonging to the same cluster reduces the chance that differences in case-mix distributions and interaction are the cause of HSMR differences across hospitals. Also for trend monitoring of HSMRs within a single hospital, the case-mix distributions of those years, and potential interaction between year and case-mix must be analysed before interpreting possible changes in HSMR.

## Supporting Information

Appendix S1 (DOCX)

Appendix S2 (DOCX)
