# Supplementary material for: Hospital Standardized Mortality Ratio: Consequences of Adjusting Hospital Mortality with Indirect Standardization
Source: PLoS One. 2013 Apr 9;8(4):e59160. doi: 10.1371/journal.pone.0059160 (PMC3621877; doi:10.1371/journal.pone.0059160)
Supplement: Appendix S1 — (DOCX) [file pone.0059160.s001.docx]

## Appendix S1

Suppose hospital A distinguishes two kinds of patients: patients admitted urgently and non-urgently admitted patients. Suppose that in year t hospital A has admitted 20% of the 5000 patients urgently. Let us now assume that urgently admitted patients have an expected mortality probability of 6% and non-urgently admitted patients have an expected mortality probability of 2% (see also table A.1). Furthermore, we assume that the *observed* mortality rates for these groups are 3% and 4% respectively.

Suppose that in year t+1 the quality of care of hospital A drops, expressed as increased observed mortality rates of 4% and 5% for urgently admitted patients and for non-urgently admitted patients respectively (see table A.1). Furthermore the number of urgently admitted patients and non-urgently admitted patients for that year changes to 2000 and 3000 respectively. This yields a HSMR of 128, which is lower than 136 in year *t* and suggests an increase in quality of care rather than a decrease. Hence, theoretically, a decreased performance of quality of care, reflected as an increase in observed mortality rates, can remain unnoticed due to differences in case-mix distribution.

Table A.1: Numerical example of HSMRs of hospital A in two consecutive years

|  | **Hospital A (year t)** | | **Hospital A (year t+1)** | |
| --- | --- | --- | --- | --- |
|  | Urgent | Non-urgent | Urgent | Non-urgent |
| **Expected mortality rate** | 6% | 2% | 6% | 2% |
| **Observed mortality rate** | 3% | 4% | 4% | 5% |
| **Case-mix** | 1000 | 4000 | 2000 | 3000 |
| **HSMR** | 136  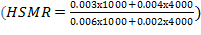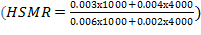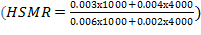 | | 128  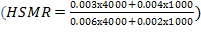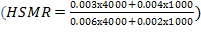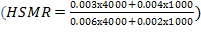 | |

*Despite a drop in quality of care, reflected as higher observed mortality rates, the HSMR improves in year t+1 because of a different case-mix distribution.*
