# Supplementary material for: Hospital Standardized Mortality Ratio: Consequences of Adjusting Hospital Mortality with Indirect Standardization
Source: PLoS One. 2013 Apr 9;8(4):e59160. doi: 10.1371/journal.pone.0059160 (PMC3621877; doi:10.1371/journal.pone.0059160)
Supplement: Appendix S2 — (DOCX) [file pone.0059160.s002.docx]

## **Appendix 2. Hospitals and their HSMRs**

**Table A.2. Scenario analysis HSMR.**

| Original | | | Scenario 1 | | | | Scenario 5 | | | |
| --- | --- | --- | --- | --- | --- | --- | --- | --- | --- | --- |
| Ranking | HSMR | | HSMR | | New ranking | Rank difference | HSMR | | New ranking | Rank difference |
| 1 | 68 | (*) | 69 | (*) | 2 | -1 | 69 | (*) | 1 | 0 |
| 2 | 69 | (*) | 68 | (*) | 1 | 1 | 73 | (*) | 2 | 0 |
| 3 | 79 | (*) | 83 | (*) | 5 | -2 | 77 | (*) | 4 | -1 |
| 4 | 79 | (*) | 79 | (*) | 3 | 1 | 76 | (*) | 3 | 1 |
| 5 | 82 | (*) | 82 | (*) | 4 | 1 | 82 | (*) | 5 | 0 |
| 6 | 85 | (*) | 84 | (*) | 6 | 0 | 86 | (*) | 7 | -1 |
| 7 | 86 | (*) | 86 | (*) | 8 | -1 | 85 | (*) | 6 | 1 |
| 8 | 87 | (*) | 86 | (*) | 7 | 1 | 91 | (*) | 16 | -8 |
| 9 | 87 | (*) | 88 | (*) | 11 | -2 | 90 | (*) | 15 | -6 |
| 10 | 88 | (*) | 88 | (*) | 10 | 0 | 87 | (*) | 8 | 2 |
| 11 | 88 | (*) | 87 | (*) | 9 | 2 | 89 | (*) | 10 | 1 |
| 12 | 89 | (*) | 90 | (*) | 13 | -1 | 88 | (*) | 9 | 3 |
| 13 | 90 |  | 90 |  | 12 | 1 | 89 |  | 11 | 2 |
| 14 | 90 | (*) | 91 | (*) | 15 | -1 | 90 | (*) | 14 | 0 |
| 15 | 91 | (*) | 91 | (*) | 14 | 1 | 92 |  | 17 | -2 |
| 16 | 91 |  | 91 |  | 17 | -1 | 90 |  | 12 | 4 |
| 17 | 92 | (*) | 92 | (*) | 18 | -1 | 92 | (*) | 18 | -1 |
| 18 | 92 |  | 91 |  | 16 | 2 | 93 |  | 19 | -1 |
| 19 | 92 |  | 92 |  | 19 | 0 | 95 |  | 23 | -4 |
| 20 | 93 |  | 94 |  | 21 | -1 | 94 |  | 21 | -1 |
| 21 | 94 |  | 93 |  | 20 | 1 | 107 |  | 44 | -23 |
| 22 | 95 |  | 95 |  | 25 | -3 | 96 |  | 25 | -3 |
| 23 | 95 |  | 96 |  | 26 | -3 | 123 | (**) | 57 | -34 |
| 24 | 96 |  | 94 |  | 23 | 1 | 96 |  | 26 | -2 |
| 25 | 96 |  | 94 |  | 22 | 3 | 95 |  | 24 | 1 |
| 26 | 96 |  | 96 |  | 30 | -4 | 94 |  | 22 | 4 |
| 27 | 97 |  | 96 |  | 29 | -2 | 96 |  | 27 | 0 |
| 28 | 97 |  | 96 |  | 28 | 0 | 97 |  | 28 | 0 |
| 29 | 97 |  | 94 |  | 24 | 5 | 90 | (*) | 13 | 16 |
| 30 | 97 |  | 96 |  | 31 | -1 | 98 |  | 30 | 0 |
| 31 | 97 |  | 96 |  | 27 | 4 | 101 |  | 35 | -4 |
| 32 | 98 |  | 97 |  | 32 | 0 | 100 |  | 32 | 0 |
| 33 | 98 |  | 98 |  | 33 | 0 | 98 |  | 29 | 4 |
| 34 | 100 |  | 100 |  | 34 | 0 | 94 |  | 20 | 14 |
| 35 | 100 |  | 100 |  | 36 | -1 | 100 |  | 33 | 2 |
| 36 | 101 |  | 102 |  | 41 | -5 | 107 |  | 46 | -10 |
| 37 | 101 |  | 100 |  | 35 | 2 | 101 |  | 34 | 3 |
| 38 | 101 |  | 101 |  | 38 | 0 | 101 |  | 36 | 2 |
| 39 | 101 |  | 101 |  | 37 | 2 | 99 |  | 31 | 8 |
| 40 | 102 |  | 103 |  | 42 | -2 | 102 |  | 37 | 3 |
| 41 | 102 |  | 102 |  | 40 | 1 | 118 | (**) | 55 | -14 |
| 42 | 102 |  | 102 |  | 39 | 3 | 104 |  | 39 | 3 |
| 43 | 103 |  | 103 |  | 43 | 0 | 104 |  | 40 | 3 |
| 44 | 103 |  | 104 |  | 46 | -2 | 107 |  | 45 | -1 |
| 45 | 104 |  | 103 |  | 44 | 1 | 103 |  | 38 | 7 |
| 46 | 105 |  | 104 |  | 45 | 1 | 104 |  | 41 | 5 |
| 47 | 105 |  | 105 |  | 48 | -1 | 105 |  | 42 | 5 |
| 48 | 105 |  | 105 |  | 47 | 1 | 106 |  | 43 | 5 |
| 49 | 107 |  | 107 |  | 49 | 0 | 114 | (**) | 52 | -3 |
| 50 | 108 |  | 108 |  | 51 | -1 | 108 | (**) | 47 | 3 |
| 51 | 109 |  | 108 |  | 52 | -1 | 113 | (**) | 51 | 0 |
| 52 | 109 |  | 109 |  | 53 | -1 | 110 |  | 49 | 3 |
| 53 | 110 |  | 107 |  | 50 | 3 | 110 |  | 48 | 5 |
| 54 | 110 | (**) | 110 | (**) | 54 | 0 | 110 | (**) | 50 | 4 |
| 55 | 114 | (**) | 114 | (**) | 56 | -1 | 129 | (**) | 60 | -5 |
| 56 | 115 |  | 114 |  | 55 | 1 | 125 | (**) | 58 | -2 |
| 57 | 120 | (**) | 121 | (**) | 58 | -1 | 114 | (**) | 53 | 4 |
| 58 | 120 | (**) | 120 | (**) | 57 | 1 | 117 | (**) | 54 | 4 |
| 59 | 122 | (**) | 125 | (**) | 59 | 0 | 123 | (**) | 56 | 3 |
| 60 | 128 | (**) | 128 | (**) | 60 | 0 | 129 | (**) | 59 | 1 |
| 61 | 132 | (**) | 132 | (**) | 61 | 0 | 155 | (**) | 61 | 0 |

*The HSMR of scenario 1 is computed based on the mean of the case-mix distributions of the ‘urgency of admission’ variable of the 61 hospitals. The HSMR of scenario 5 is computed based on the mean of the case-mix distributions of the ‘Charlson Co morbidity index’ variable of the 61 hospitals. (*) Significantly lower than 100, (**) significantly higher than 100.*
